# A scoping review of the methodological quality of research on mental health of healthcare professionals in low- and lower-middle income countries

**DOI:** 10.12688/wellcomeopenres.17916.2

**Published:** 2023-07-14

**Authors:** Julia Lohmann, Denny John, Aso Dzay

**Affiliations:** 1 Department of Global Health and Development, London School of Hygiene & Tropical Medicine, London, WC1H 9SH, UK; 2 Heidelberg Institute of Global Health, University Hospital Heidelberg, Heidelberg, 69120, Germany; 3 Faculty of Life and Allied Health Sciences, Ramaiah University of Applied Sciences, Bengaluru, 560054, India; 4 Evidence Synthesis and Implementation for Indigenous Health: A JBI Affiliate Centre, Centre for Public Health Research (CPHR), MANT, Kolkata, 700078, India

**Keywords:** Mental health, psychological wellbeing, health workers, low- and lower-middle-income countries, methodological quality

## Abstract

**Background:** SARS-CoV-2 has resulted in widespread awareness of health workers’ work realities and their mental health impacts, and corresponding unprecedented research effort. Reviews of the quantitative literature on mental health of clinical skilled healthcare personnel in low- and lower-middle income countries (LLMIC), however, point at quality issues in the pre-pandemic literature. We used the evidence generated in the context of one pre-pandemic review to understand methodological strengths and weaknesses in detail, with the aim of distilling recommendations for future research.

**Methods:** Our study used the literature identified in a systematic search from inception to the end of 2020, in English or French language, in MEDLINE, EMBASE, PsychINFO, Global Health, and CAIRN. Following a scoping review approach, we extracted and charted data on key study characteristics as well as on study quality. In regard to the latter, we developed nine quality criteria on the basis of existing quality checklists, but expanding on issues of particular relevance to the measurement and interpretation of levels of mental health or illness. We collated the charted data in descriptive fashion.

**Results:** We included data from 152 studies, which assessed a range of mental health outcomes, although most burnout. Most studies were conducted in India, Nigeria, Pakistan, or Egypt, in urban secondary- and tertiary-care settings. We judged only 20% of studies as of high quality due to shortcomings particularly regarding sample representativeness, context-specific measurement tool validity, and reporting of methodological detail.

**Conclusion:** We conclude that despite its impressive size, we can learn comparatively little from the body of literature up to the end of 2020 due to narrow study focus on specific settings and strong limitations in quality. Based on our findings, we outline areas for expansion, methodological improvement, and standardization of reporting in future research.

**PROSPERO Registration: **CRD42019140036.

## Abbreviations

CASP        Critical Appraisal Skills Programme

DASS        Depression, Anxiety and Stress Scale

DSM         Diagnostic and Statistical Manual of Mental Disorders

GHQ         General Health Questionnaire

HIC           High-income countries

ICD           International Statistical Classification of Diseases and Related Health Problems

JBI            Joanna Briggs Institute

LLMIC     Low- and lower-middle income countries

MBI-HSS Maslach Burnout Inventory - Health Services Survey

NIH          National Institutes of Health

PHQ         Patient Health Questionnaire 

RPA         Reduced personal accomplishme

## Background

The SARS-CoV-2 pandemic has painfully reminded the global health community of the difficult and often precarious conditions among which health care professionals all over the world work to avert illness and deaths, not only in times of crisis, but also in “normal” times, placing their physical and mental health at substantial risk.

Pre-pandemic research on health worker mental health and psychological wellbeing is widely available for high-income countries (HIC) [e.g. 1– 4], demonstrating often alarming levels of burnout and other mental health conditions, identifying a large number of determinants, and linking poor mental health to adverse consequences for patients and the health system at large. In non-HIC settings, however, several pre-pandemic reviews have evaluated the evidence base on the mental health situation among health workers as limited ^5– 9^. The pandemic has generated a surge in research – and associated reviews – on mental health issues among health workers ^10^, albeit similar to pre-pandemic work with a strong focus on high- and upper-middle-income settings.

Beyond limitations in scope of research available from low- and lower-middle-income countries (LLMIC), reviews point at limitations in quality ^5– 9^. Quality assessments, if done, were performed at a rather high level of abstraction in line with the standard for systematic reviews, but do not go into the level of detail that would allow understanding concrete areas for methodological improvement. For instance, Chemali and colleagues ^5^ and Dubale and colleagues ^6^, in reviewing the health worker burnout literature in the Middle East and sub-Saharan Africa, respectively, used the Newcastle-Ottawa Scale to assess quality of the predominant cross-sectional included studies, which similar to other standard quality assessment tools does not go into much detail on issues of measurement. They further only list quality assessment results at the highest aggregation level, but do not discuss them in much depth beyond how lack of local validation studies and inconsistent use of the Maslach Burnout Inventory (MBI) as the dominant measurement tool complicate the interpretation of study findings. Similarly, Dugani and colleagues ^7^ and Kesarwani and colleagues ^8^ highlight measurement and sample size issues and other representativeness issues, without having assessed study quality in detail, however.

Our study aims to address this research quality knowledge gap by building on a systematic review of the quantitative literature on mental health and psychological wellbeing of clinical skilled healthcare personnel working in all settings of care in LLMIC worldwide ^9, 11^. Specifically, the quality issues highlighted by this and prior similar reviews ^5– 8^ inspired efforts to return to the identified literature to undertake a more detailed quality assessment, with a focus on the measurement of prevalences of mental illness, to generate an in-depth understanding of the methodological strengths and weaknesses and to distil concrete recommendations as to how research could be improved in the future. Our works is aimed at researchers, funders, and policy makers working towards building a high-quality evidence base to inform improvement of the psychological wellbeing of the healthcare workforce. 

## Methods

### Study design

The analysis reported in this manuscript was conducted under the umbrella of a systematic review of the literature on mental health and psychological wellbeing of clinical skilled healthcare personnel working in all care settings in LLMIC worldwide, up to the end of 2020. The protocol for the systematic review was registered in the International Prospective Register of Systematic Reviews (PROSPERO) (registration number: CRD42019140036) and has been published ^11^. Results of the systematic review are reported elsewhere ^9^.

This manuscript reports the results and recommendations arising from additional data extraction and analysis pertaining to methodological quality as outlined in the introduction. Given the somewhat ambiguous nature of the study – study identification and selection took place in the context of a systematic review, but data extraction and analysis diverged from the core systematic review and its research aims –, we adopted a scoping review approach to analyzing and reporting the findings ^12^. We followed the framework by Arksey and O’Malley ^13^, modified by Levac, Colquhoun and O’Brien ^14^, as well as the essential reporting items as proposed by the PRISMA extension for scoping reviews ^15^ (see Extended Data, Additional Files 1 ^16^), in writing the manuscript.

An earlier version of this article can be found on Research Square (doi: https://doi.org/10.21203/rs.3.rs-215108/v1).

### Study identification

We performed a systematic search of the literature on mental health and psychological wellbeing of clinical skilled healthcare personnel working in all care settings in low- and lower-middle income countries worldwide, from inception to the end of 2020. The search was initially conducted in June 2019 as part of an MSc project ^9, 11^. In the context of the quality assessment reported in this manuscript, it was then updated in June 2021 to reflect research published until end 2020. The employed eligibility criteria, information sources, and search strategy are detailed in the following.

Eligibility criteria

**Participants:** We considered studies referring to:

- Formally and fully trained health professionals and health associate professionals ^17, 18^, specifically medical doctors, nursing and midwifery professionals, and nursing and midwifery associate professionals;
- Working in formal health care facilities (public, private not-for-profit, private for-profit);
- Working in low- and lower-middle income countries as per the World Bank’s 2019 classification ^19^ (see also Extended Data, Additional Files 2 ^16^)

We excluded studies focusing exclusively on non-clinical or not formally or not yet fully trained personnel as well as on exclusively community-based personnel (e.g., pure management or administrative personnel, traditional or lay health workers, community health workers, students and other health workers in training) due to enormous heterogeneity in cadres and terminology across countries; studies with an exclusive focus on non-LLMIC; and studies on migrant health workers from LLMIC to HIC.

**Concept:** We considered studies on burnout, depression, anxiety, trauma, general psychological wellbeing and/or distress, as well as other specific mental health/distress diagnoses or concepts if work-related and explicitly framed as a mental health issue. We considered all studies labelled by the authors as investigating the above, irrespective of whether they referred to an international disease classification system (such as ICD-10/11 or DSM-VI/V) and irrespective of the measurement tool used. We did not consider studies on stress, job or life satisfaction without specific reference to mental health.

**Context:** We considered studies conducted with health workers working in formal health care settings (public, private not-for-profit, private for-profit) in low- and lower-middle income countries as per the World Bank’s 2019 classification ^19^.

**Study designs:** We included all relevant observational and intervention, cross-sectional or longitudinal, study designs published in English or French language based on the languages familiar within the study team. Where multiple papers were generated from the same data looking at the same outcome, only the most relevant/recent paper was included. However, if multiple papers were generated from the same data with different outcomes or on different subpopulations, all papers were included.

We did not include qualitative studies, previously published systematic reviews (although we cross-checked included articles), opinion pieces, commentaries, policy briefs, and conference abstracts without identifiable fulltext.

Information sources and search strategy

We searched for eligible studies published from inception to December 2020, in English or French language, in MEDLINE, EMBASE, PsychINFO, Global Health, and CAIRN.

The search strategy included a combination of subject terms and free-text terms from three categories based on the inclusion and exclusion criteria: (1) geographic focus: all LLMIC as well as overarching regional terms; (2) population: generic terms for healthcare professionals as well as terms for specific health worker cadres; and (3) outcomes: specific terms for burnout, depression, and psychological wellbeing, generic terms for mental health/illness and work-related psychological stress/distress/trauma, and terms for specific common measurement instruments. Regarding the latter, we also included “motivation” and “satisfaction” as search terms, based on our experience that studies labelled as such sometimes contain mental health measures as part of the motivation or satisfaction measurement tool. The search terms are provided in Table 1. We customized the exact search syntax for each data base according to their specific requirements and functions, including for instance relevant MeSH terms. We tested the search strategy by including or removing terms to understand if this would yield different results.

**Table 1.** Search terms.

Eligibility criterion	Search terms
Geographic focus	Low-income countries, middle-income countries, lower-middle income countries, low-and middle income countries; all LLMIC by country name
Study population	Health/healthcare personnel, health/healthcare professional, health/healthcare worker, healthcare provider, healthcare staff, primary care provider, outpatient healthcare/health care provider, hospital nursing staff, general practitioner, allied health personnel, community health officer, community health extension worker, physician, nurse, clinician, medical assistant, clinical officer, emergency department staff, hospitalist, medical staff, nursing staff, birth attendant, midwife, pharmacist, dentist, physical therapist, physiotherapist,
Mental health outcomes – generic	Mental health (health, hygiene), motivation, stress (occupational, work, psychological), fatigue (mental, compassion), distress (mental, psychological), wellbeing (mental, psychological), job satisfaction, work-life balance
Mental health outcomes – syndromes	Burnout (psychological, professional), emotional exhaustion, depersonalization, personal accomplishment, depression, anxiety, trauma
Mental health outcomes – measures *	WHO-5 wellbeing/well-being index, Warwick Edinburg mental well-being scale

* we did not include any of the standard measures for common syndromes (burnout, depression, anxiety) in the search as they all include the respective syndrome in the title

### Study selection

**Figure 1.**
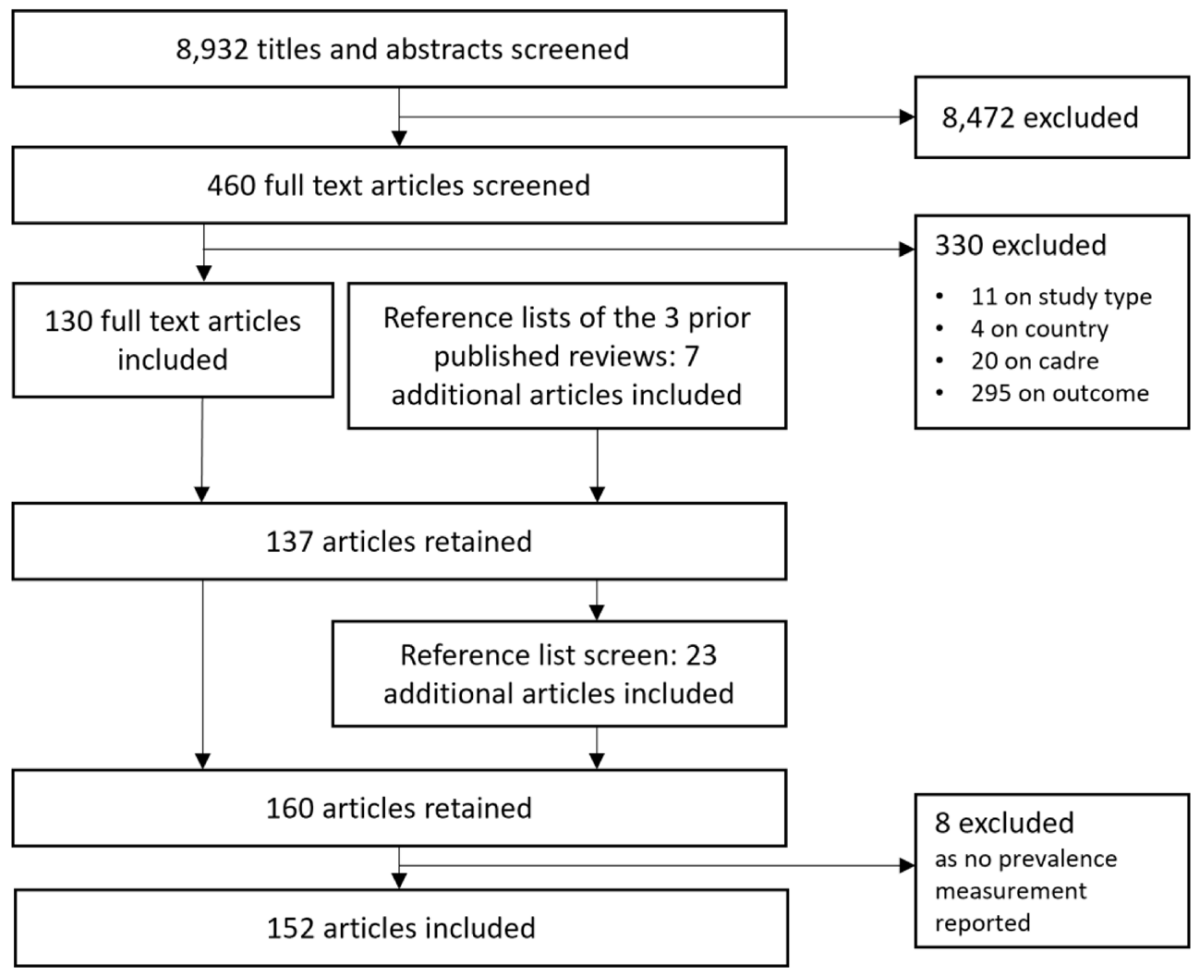
Flow chart outlining the study search and selection process.

Given the purposively broad search strategy, the search resulted in a total of 8,932 unique studies after removal of duplicates. AD and JL independently examined titles and abstracts of a subset of the studies against the eligibility criteria, comparing and discussing results until convergence. The remainder of the study titles and abstracts were screened by a single researcher. We retained and retrieved 460 studies for full text screening. The high number of initial search results compared to retained studies was largely due to the search algorithm picking up studies on mental health of non-health workers, which however made mention of treatment and thereby the health workforce in the abstract. Full-text screening was performed in full double screening by AD and JL. Discrepancies were minimal and resolved in discussion. 130 studies were retained following this stage, whereas 330 were excluded. Of the latter, the vast majority were studies on motivation and satisfaction, which we had retained for the full text screening to ensure they do not include “hidden” psychological wellbeing measures. The remaining studies were excluded because they measured non-specific stress or substance abuse (usually not clearly marked as mental health problem and including e.g. smoking), were qualitative studies, were exclusively conducted in an upper-middle or high-income country, or included only community health workers, trainees or students, or other health worker groups outside our inclusion criteria. Despite the broad search strategy, seven relevant articles included in prior systematic reviews ^5– 8^ were not picked up by our search and manually included. A screening of the reference lists of the 137 resulting articles led to the inclusion of a further 23 studies. In a final step for the purpose of the additional quality assessment presented in this manuscript, eight studies were excluded as they only reported associations of mental health with other factors, but no prevalence measurements as such. Figure 1 outlines the study search and selection process.

### Data charting

From each included study, we extracted the information summarized in Table 2 into an Excel-based data extraction form developed ex-ante. JL and AD independently extracted data from a subset of studies and compared and discussed results until reaching convergence. Data extraction was then continued by a single researcher, as full double extraction would have not been feasible within the scope of the project given the large number of studies.

**Table 2.** Extracted data elements.

Category	Data elements
Basic study information	Journal, year of publication, year of data collection, study country(ies), mental health outcome(s) measured, study type (levels vs associated factors assessed)
Study setting and population	Multiple vs single region/city/facility, number of health facilities, level of care, sector (private, public), urban vs rural setting, health worker cadres, further information on inclusion and exclusion criteria; adequacy of the description of study setting and population
Study sample	Sampling entry point (health care facilities vs other), sampling strategy, reasons for sample size, response rate, actual sample size (overall, by cadre, by gender), basic sample characteristics
Mental health outcome(s)	Measurement tool name, tool details (number of items, response scale incl numeric representation of anchors) and references provided, translation, pretest, reliability, validity, reporting of findings (proportions, continuous), calculation of composite scores, categorization into good/poor mental health groups, relevant analytical details (eg adjustment for sample composition), clarity of results tables and description

In relation to the study aim of highlighting methodological strengths and weaknesses, we then used the extracted information to evaluate the included studies assessing levels of mental health regarding nine quality criteria pertaining to the availability of information on and adequacy of basic study characteristics, study population and sample, outcome measurement, and reporting of results. The quality criteria are listed in Table 3 and Table 4 and explained in the respective results sections. In the absence of specific quality checklists for mental health prevalence studies, the quality criteria were developed by adapting and expanding commonly used quality checklists for systematic reviews, notably the Joanna Briggs Institute’s (JBI) Checklists for Analytical Cross-Sectional Studies and for Prevalence Studies ^20^, the Critical Appraisal Skills Programme’s (CASP) Case Control Study Checklist ^21^, and the National Institutes of Health’s (NIH) Quality Assessment Tool for Observational Cohort and Cross-Sectional Studies ^22^.

**Table 3.** Quality criteria.

Basic study information		
1	Is the year of data collection mentioned?	2 points: Mentioned 0 points: Not mentioned
2	Is the study setting adequately described?	Should include geographic scope, number of health facilities (if level of sampling), and setting (urban/rural) 2 points: fully adequate 1 point: mostly adequate, with only some ambiguity 0 points: insufficient
Study population and sample		
3	Is the study population clearly specified and defined?	Should include level of care, sector (public/private), health worker cadre and specialization (if applicable) 2 points: clear description 1 points: mostly clear, with only some ambiguity 0 points: unclear
4	Based on the description of the sampling strategy and outcome, is the sample likely to be representative for the intended study population?	2 points: highly likely (convincing description of census or random sample with response rate > 70%) 1 point: somewhat likely (convincing description of census or random sample with response rate 50-70% or not provided; well-described and convincing convenience sampling) 0 points: unlikely (convenience sample, unconvincing description of a “declared” census or random sample, insufficient information about the sampling strategy to judge)
5	Are the study participant characteristics described in sufficient detail?	Should allow for comparability with another study conducted in the setting, and include at minimum sex, age or seniority in health care, and health worker type/ cadre; numbers and proportions should add up 2 points: clear description 1 points: largely clear, with only some ambiguity/omissions and/or inconveniences in display 0 points: unclear or containing obvious mistakes that cannot be “recalculated by hand”
Outcome measurement		
6	Do the authors adequately report the tool(s) used to measure the outcome(s)?	If established measure: should include name, version (if multiple), and language of tool (if no official translation), including any modifications; for non-established tools: should include clear description including item list, response modalities, and scoring, OR reference in the public domain including clear description 2 points: fully adequate 1 point: largely sufficient, with only some ambiguity 0 points: insufficient
7	Do the authors report convincing information on the validity of the tool(s) used to measure the outcome(s)?	Ideally, this should include information on reliability as well as content, structural, and criterion validity relevant to the context; if proportions are reported, should include information of the validity of the threshold(s) used for classification in the context; references to appropriate validation papers in the public/academic domain are acceptable 2 points: convincing and rich validity information (ie. content/criterion validity from the context plus threshold validity if proportions are reported) 1 points: some, but incomprehensive or not fully convincing validity information (e.g. threshold validity missing, but good info on content/criterion validity; only alphas but no other content/criterion validity reported if measures only used continuously) 0 points: no or unconvincing validity information (eg no validation in the context, application of “standard” thresholds)
8	Do the authors provide all necessary background information to interpret numeric representations of measurements?	Should include all info necessary to interpret measurements, so on response scale, aggregation, categorization, thresholds, etc. For established tools, clear reference to tool manual for details is acceptable. 2 points: fully adequate 1 point: largely sufficient, with only some ambiguity about which assumptions can be made with reasonable certainty (eg that an established tool was used in its standard form) 0 points: inadequate, does not allow to interpret the measurements even with some assumptions
Reporting		
9	Are results adequately displayed?	1: results sufficiently conveniently displayed without apparent errors 0: results displayed in a way that necessitates guesses and/or with clear errors

**Table 4.** Quality classification.

High quality	a) 14 or more quality points, AND b) Results reported in readable manner (i.e. 1 point on criterion 9), AND c) Sufficient key information provided to allow for the measurements to be interpreted (i.e. 2 points on criteria 6 and 8 *)
Moderate quality	a) 11 – 13 quality points OR b) > 13 quality points, but not fulfilling criteria b) and/or c) for high quality
Low quality	0 – 10 quality points

* with the exception of MBI-22 (HSS) studies which did not specify the exact thresholds used to categorize respondents. We scored these with 1 on quality criterion 8 for consistency with other studies. However, differences in the various thresholds circulating in the academic literature are minimal, so that they are unlikely to distort prevalence estimates to non-comparability.

While we borrowed heavily from these checklists, our quality criteria contain more detail on measurement and validity aspects in line with the specific importance of these aspects in the measurement of mental health constructs as explained in the introduction.

JL and AD independently evaluated a subset of studies on the quality criteria and compared and discussed results until reaching common understanding indicated by convergence in assessment. The resulting studies were assessed by only one researcher.

### Collating, summarizing and reporting of the results

In a final step, we summarized and organized the extracted data by quality category. We included in the evaluation of methodological quality both studies with an explicit study aim to assess mental health levels, as well as studies where estimation of mental health levels was a by-product of a study with a different primary aim. Acknowledging that a less strict benchmark is appropriate for the latter category of studies, we will present results separately for both groups. All analysis was descriptive in line with the descriptive study aim to describe methodological strengths and weaknesses of the literature.

## Results

**Figure 2.**
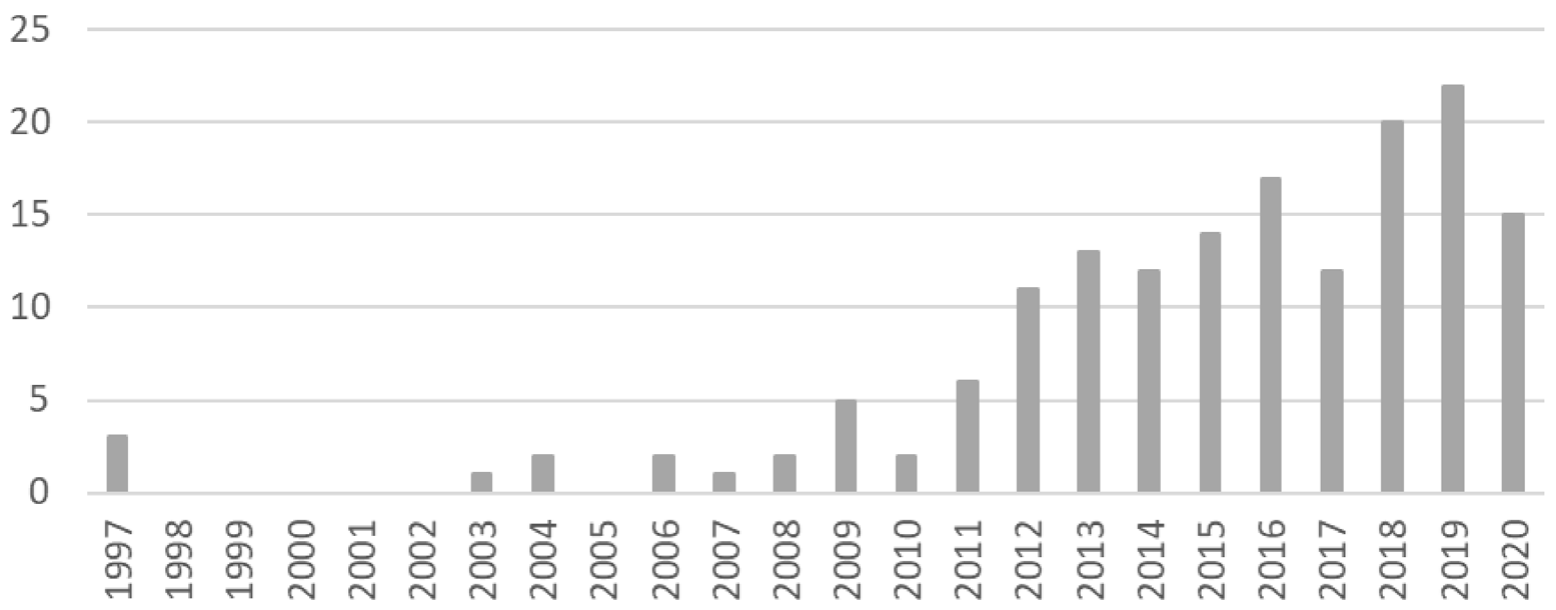
Included studies by year of publication.

The search and selection process ( Figure 1) resulted in a total 152 articles reporting levels of good or poor mental health or psychological wellbeing among clinical, skilled healthcare personnel in LLMIC published up to December 2020. A full list of the included articles as well as an overview over key characteristics is provided in the Extended Data (Additional Files 3) ^16^. There is a clear increase in availability of studies over time, with half of the included articles being published in 2016 or later and less than 5% before 2006 ( Figure 2). Of note, 38% of studies did not report the year in which data collection took place ( *quality criterion 1*).

In the following, we integrate an overview of key characteristics of the included studies ( Table 5) with findings of the quality assessment ( Table 6), as both are inextricably linked. We begin with study countries and settings, followed by study populations and samples, study outcomes and measures, and results reporting, before closing with an analysis of overall study quality.

**Table 5.** Key study characteristics.

	All studies	Prevalence study subset
**Total number of studies**	**152**	**92**
**Study region**		
WHO Africa Region	60 (39.5 %)	38 (41.3 %)
WHO Eastern Mediterranean Region	45 (29.6 %)	25 (27.2 %)
WHO South-East Asia Region	38 (25.0 %)	24 (26.1 %)
WHO Western Pacific Region	9 (5.9 %)	5 (5.4 %)
Study setting *		
Urban	131 (86.2 %)	82 (89.1 %)
Rural	41 (27.0 %)	23 (25.0 %)
Single-site	50 (32.9 %)	34 (37.0 %)
Study population *		
Primary level of care	38 (25.3 %)	22 (23.9 %)
Second and/or tertiary level of care	143 (95.3 %)	88 (95.7 %)
Medical doctors	83 (53.3 %)	56 (60.9 %)
Nurses	109 (71.7 %)	60 (65.2 %)
Other clinical staff	43 (28.9 %)	28 (30.4 %)
Other managerial staff	5 (3.3 %)	4 (4.4 %)
**Sampling**		
Census	59 (38.8 %)	44 (47.8 %)
Random / stratified random sample	26 (17.1 %)	17 (18.5 %)
Convenience sample	39 (25.7 %)	19 (20.7 %)
Unclear	28 (18.4 %)	12 (13.0 %)
Study outcomes *		
Burnout	97 (63.8 %)	61 (66.3 %)
Depression	16 (10.5 %)	11 (12.0 %)
Anxiety	13 (8.6 %)	8 (8.7 %)
Trauma	6 (4.0 %)	3 (3.4 %)
General psychological wellbeing	40 (26.3 %)	22 (23.9 %)
Outcome measurement *		
Continuous outcome	86 (56.6 %)	41 (44.6 %)
Proportions / categorical outcome	98 (64.5 %)	78 (84.8 %)
**Results reporting**		
By cadre: yes, by design	97 (63.8 %)	57 (62.0 %)
By cadre: yes	24 (15.8 %)	18 (19.5 %)
By cadre: no	31 (20.4 %)	17 (18.5 %)
By gender: yes, by design	14 (9.2 %)	6 (6.5 %)
By gender: yes	55 (36.2 %)	42 (45.7 %)
By gender: no	83 (54.6 %)	44 (47.8 %)

*Proportions do not add up to 100% as many studies include mixed settings/study populations and/or several outcomes

**Table 6.** Study quality by study type.

	All studies	Prevalence study subset
Qual 1: Is the year of data collection mentioned?		
2 Mentioned	95 (62.5 %)	63 (68.5 %)
0 Not mentioned	57 (37.5 %)	29 (31.5 %)
Qual 2: Is the study setting adequately described?		
2 Fully adequate	128 (84.2 %)	80 (87.0 %)
1 Mostly adequate, with only some ambiguity	13 (8.6 %)	6 (6.5 %)
0 Insufficient	11 (7.2 %)	6 (6.5 %)
Qual 3: Is the study population clearly specified and defined?		
2 Fully clear	138 (90.8 %)	87 (94.6 %)
1 Mostly clear, with only some ambiguity	13 (8.6 %)	5 (5.4 %)
0 Unclear	1 (0.6 %)	0 (0 %)
Qual 4: Is the sample likely to be representative for the intended study population?		
2 Highly likely	40 (26.3 %)	24 (26.1 %)
1 Somewhat likely	48 (31.6 %)	38 (41.3 %)
0 Unlikely	64 (42.1 %)	30 (32.6 %)
Qual 5: Are the study participant characteristics described in sufficient detail?		
2 Fully adequate	117 (83.6 %)	80 (87.0 %)
1 Mostly adequate, with only some ambiguity	17 (11.2 %)	9 (9.8 %)
0 Insufficient	8 (5.2 %)	3 (3.2 %)
Qual 6: Is the tool(s) used to measure the outcome(s) adequately reported?		
2 Fully adequate	131 (86.2 %)	81 (88.0 %)
1Mostly adequate, with only some ambiguity	11 (7.2 %)	8 (8.7 %)
0 Insufficient	10 (6.6 %)	3 (3.3 %)
Qual 7: Is convincing information on the validity of the tool(s) used to measure the outcome(s) reported?		
2 Convincing	12 (7.9 %)	6 (6.5 %)
1 Partially convincing	42 (27.6 %)	15 (16.3%)
0 Unconvincing or no information provided	98 (64.5 %)	71 (77.2 %)
Qual 8: Is all necessary background information to interpret numeric representations of measurements provided?		
2 Convincing	86 (56.6 %)	50 (54.4 %)
1 Partially convincing	44 (29.0 %)	32 (34.8%)
0 Unconvincing or no information provided	22 (14.4 %)	10 (10.8 %)
Qual 9: Are results adequately displayed?		
1 Adequate	141 (92.8 %)	84 (91.3 %)
0 Unclear or containing obvious errors	11 (7.2 %)	8 (8.7 %)
**Combined quality classification**		
High quality	30 (19.7 %)	19 (20.6 %)
Moderate quality	89 (58.6 %)	57 (62.0 %)
Low quality	33 (21.7 %)	16 (17.4 %)

Study country and setting

**Figure 3.**
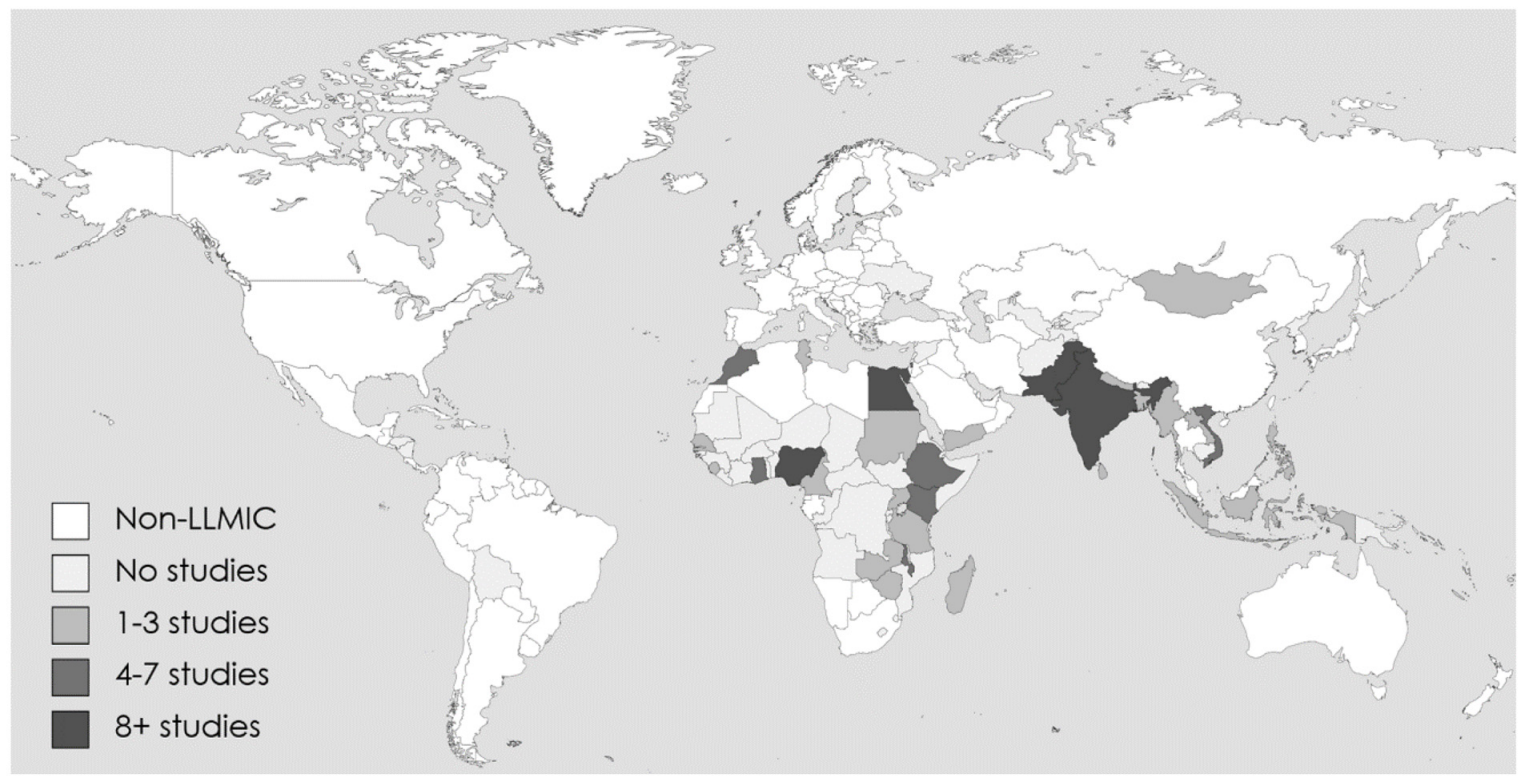
Geographic distribution of studies.

**Study country.** The 152 studies cover a total of 29 unique countries ( Figure 3).

Geographically, in alignment with where most LLMIC are located, most studies (40%) were conducted in countries of the WHO Africa Region. LLMIC in the WHO European Region and Region for the Americas are not represented in the included studies. Close to two thirds of studies were conducted in four countries, namely India (32 studies), Nigeria (27 studies), Pakistan (17 studies), and Egypt (12 studies). The number of studies in each of the remaining 25 countries ranged between 1 and 7.

**Study setting.** 65% of studies were conducted in urban settings, 6% in rural settings, 21% in both, and for 8%, it was not possible to judge based on the reported information. 90 studies (59%) were conducted within only one city, of which 50 studies within only one healthcare facility, most of which university or other tertiary care hospitals. For multi-site studies, the number of healthcare facilities from which respondents were sampled ranged from 2 to 89 (mean=11.9, sd=17.3), with 14% of studies not reporting and 13% of studies having sampled through other channels.

Reporting of the study setting ( *quality criterion 2*) was largely satisfactory, with 84% of studies reporting sufficient information. Among explicit prevalence studies, reporting was slightly better than overall. 

Study populations and samples

**Study population.** 95% of study samples included health workers working at secondary- and/or tertiary-level health facilities, of which 43% exclusively tertiary-level staff. Only 25% of study samples, from a total of 16 countries, included health workers at the primary level of care. Two studies did not provide information.

We classified study populations into medical doctors, nurses, other clinical staff, and other managerial staff. 64% of studies investigated only one staff category, whereas the remaining 36% two or more. 53% of studies included medical doctors, 72% nurses, 29% other clinical staff, and 3% other managerial staff. 

Overall, 91% of studies reported fully adequate information on the study population ( *quality criterion 3*; 95% in prevalence studies).

**Sampling and resulting samples.** 86% of studies sampled respondents using multi-step procedures, where they first selected health facilities, some then specific departments within health facilities, and then respondents. Some studies had further explicit pragmatic inclusion criteria such as only staff who had been working at the facility for a specific time frame, or inclusion criteria related to the main study aim, such as only respondents who had witnessed death recently. Only 14% of studies sampled respondents directly, for instance by making use of mailing lists and meetings of professional associations, or by snowballing from the researchers’ own networks.

39% of studies described their sampling strategy as a census, 17% as a random or stratified random sample, and 26% as a convenience sample. For 18% of studies, the sampling strategy was unclear (13% among prevalence studies). The proportion of studies with a census or random/stratified random sample was substantially higher among explicit prevalence studies than among studies with a different primary aim (66% vs. 40%).

Among the 26 studies with a declared random or stratified random sample, 42% provided a rationale for the envisioned sample size (41% of prevalence studies). 21 studies (81%) provided a response rate (88% of prevalence studies). 17 of the 21 studies reported a response rate above 70% (12 of the 15 prevalence studies). It is important to note, however, that 8 studies reported response rates of 98% or higher, calling into question whether the studies really used a fully random sample as opposed to some elements of replacement and/or convenience.

Of the 39 studies with a declared convenience sample, 8 provided a rationale for the envisioned sample size, 14 did not, and 17 did not state which sample size they attempted to reach at all.

Among the 59 studies with a declared (attempted) census, 47 (80%) provided a response rate (82% of explicit prevalence studies). 30 of the 47 studies reported a response rate above 70% (20 of the 35 prevalence studies).

Resulting sample sizes ranged from 29 to 2245 respondents (mean= 284.8, sd= 283.7). The difference in sample size between prevalence studies and studies with a different primary aim was small (mean 277.5 vs. 296.2, not statistically significant).

Based on the description of the sampling strategy and resulting sample ( *quality criterion 4*), we judged only 26% of studies to be based on a sample highly likely to be representative of the intended study population (also 26% of prevalence studies).

Of note, as most studies sampled health workers through health facilities and relied on health workers present at work, they by design did not capture health workers ill enough not to be able to work, making them prone to a systematic underestimation of severe cases of mental illness. This was discussed and acknowledged as a limitation by only a handful of studies.

Reporting of key respondent characteristics ( *quality criterion 5*), defined as at minimum sex, age and/or seniority in health care, and health worker type or cadre, was fully adequate in 84% of studies (87% among prevalence studies), mostly adequate with only some ambiguity or omission in 11% of studies, and insufficient in 5% of studies.

Study outcomes and measures

**Study outcomes:** 88% of studies assessed only one mental health outcome, 11% two outcomes, and 1% three outcomes. 64% of studies assessed burnout, 11% depression, 9% anxieties, 4% traumata, and 26% general psychological wellbeing.

**Outcome measures:** All burnout studies used self-reported measures rather than diagnostic interviews. Among the 97 burnout studies, the Maslach Burnout Inventory - Health Services Survey (MBI-22 HSS) was by far the most common measurement tool, used by 59 studies (61%). A further 14 studies used adaptations of the MBI (e.g., only one subscale, only selected items) or unspecified MBI versions. 8 studies (8%) used the Copenhagen Burnout Inventory, either in full or one of the subscales. The remaining 16 studies used other established or self-developed tools including the Oldenburg Burnout Inventory (4 studies); a two-item measure developed by Mbindyo *et al.* ^23^ as part of a motivation inventory (4 studies); PROQoL (2 studies); the Shirom Melamed Burnout Inventory (1 study); the Freudenberger Burnout Scale (1 study); three self-developed items (1 study); the Standard Compassion Fatigue Self-Test (1 study); or a single-item direct question (“Do you feel burned out?”; 2 studies).

Among the 16 studies having assessed depression, only one used a clinical interview (depression component of the Structured Clinical Interview for DSM-IV), while the remaining 15 studies used self-reported measures, including the Depression, Anxiety and Stress Scale (DASS-21; 5 studies); the Aga Khan University Anxiety and Depression Scale (2 studies); the Patient Health Questionnaire (PHQ-9; 2 studies); the Zung Depression Scale (2 studies); the Standardized Hospital Anxiety and Depression Scale (2 studies); the Beck Depression Inventory (1 study); and the Death Distress Scale (1 study).

Similar to depression, only 1 of the 13 studies assessing anxiety used a clinical interview (anxiety component of the Structured Clinical Interview for DSM-IV), while the remaining 12 studies used self-reported measures. The latter included the DASS-21 (5 studies); the Standardized Hospital Anxiety and Depression Scale (2 studies); the Aga Khan University Anxiety and Depression Scale (1 study); the Spielberger State-Trait Anxiety Inventory (1 study); the Zung Anxiety Scale (1 study); the Generalized Anxiety Disorder Scale (GAD-7; 1 study); and the Death Distress Scale (1 study).

All 6 studies assessing trauma used self-reported measures, including the Impact Event Scale – Revised (3 studies); PROQoL (2 studies); and the PTSD Checklist (1 study).

Finally, in the absence of clinical interviews for general (ie diagnosis-unspecific) psychological wellbeing, all 40 studies used self-reported tools, including the General Health Questionnaire (GHQ) in the 12-item (12 studies), the 28-item (4 studies), the 30-item (5 studies), or an unspecified (1 study) version; the Warwick Edinburgh Mental Wellbeing Scale (4 studies); the WHOQOL-BREF (6 studies); the WHO-5 Wellbeing Index (2 studies); Ryff's Psychological Well-being Scales (2 studies); the Kessler Psychological Distress Scale (K6; 1 study); the SF-36 mental health subscale (1 study); the SRQ-20 (1 study); the Reker Wong Perceived Wellbeing Scale (1 study); and an unspecified tool (1 study).

We considered reporting of the tool(s) to measure outcome(s) adequate ( *quality criterion 6*) if name, version, language, and any potential modifications were clearly reported or referenced. In the case of non-established tools, we expected a clear description including the item list and response modalities. 86% of studies met our criteria (88% of prevalence studies), whereas 14% of studies either reported with some ambiguity or insufficiently.

Of note, only few articles demonstrate awareness of the limitations and implications associated with using a self-reported tool rather than a clinical interview to measure the mental health outcome.

**Validity considerations:** Given the culture-sensitive nature of mental health and the predominant use of self-reported measures, we further assessed the extent to which studies provided convincing information of the validity of the tool used to measure the intended mental health constructs ( *quality criterion 7*). We considered validity information as convincing if the study provided self-generated content and criterion validity (e.g. a convincing combination of expert judgement/qualitative pre-study, Confirmatory Factor Analysis, and assessment of relationships with related constructs) which, based on the description, was achieved following standard psychometric quality criteria and yielded adequate psychometric results, or if the study referred to an external validation paper which was accessible, provided similar high-quality evidence, and was carried out in a similar population (ie at minimum same country or cultural context, even if different population). For studies reporting their measurements in categorical fashion (see below), we further required context-appropriate validity evidence of the threshold used to classify respondents into different mental health categories.

Only 8% of studies provided information which we considered convincing. 27% of studies provided some, but incomprehensive or not fully convincing validity information. 65% of studies provided no or unconvincing validity information. The proportion of studies providing convincing validity information was even lower among explicit prevalence studies (convincing: 6%; somewhat convincing: 18%; insufficient: 76%), which is due to the higher proportion of studies reporting categorical outcomes and failing to provide validity evidence regarding the used thresholds to categorize respondents.

Of note, only 23% of studies reported having performed a pretest (both overall and among explicit prevalence studies). Irrespective of the quality or appropriateness of the information, 63% of studies provided some information on reliability (usually Cronbach’s alpha) and 51% of studies provided some information on validity (usually references to the tool manual and/or validation studies conducted in high-income settings). Among explicit prevalence studies, some information on reliability and validity (irrespective of quality of the information) was provided by 53% and 52%, respectively.

**Measurement.** Beyond the measured outcomes and tools themselves, studies differed in how they reported the outcome measurements. All utilized measurement tools employed either Likert response scales or symptom counts and therefore, in a first analytical step, resulted in a quasi-continuous numeric measurement. 36% of the included studies reported outcome measurements only in this “crude” metric, i.e. as means of sum scores or scale means (15% of prevalence studies). 43% of studies divided respondents into different categories along this quasi-continuous raw score, and reported only proportions of participants in each category (55% of prevalence studies). 21% of studies reported data both in quasi-continuous and in proportional form (29% of prevalence studies).

We assessed the extent to which the authors provided all necessary background information to interpret numeric representations of measurements ( *quality criterion 8*), including the numeric codes used for response options, information on aggregation, and for studies reporting proportions, information on thresholds for categorization. 57% of studies reported sufficient information to allow interpretation and comparison to other studies having used the same measurement tool (54% of prevalence studies). For 29% of studies, there was some ambiguity, about which reasonable assumptions can be made however (35% of prevalence studies). For 14% of studies, information was insufficient (11% of prevalence studies). 

Given the widespread use of the MBI-22, we wish to highlight three specific issues frequently encountered and complicating comparability of results across studies. First, how to use the MBI is rather strictly prescribed by the publishers. However, many studies did not adhere to the publisher’s prescription, rather having altered certain items, used different response scales (number of answer options; numeric representation of answer options; labelling of answer options), leading to different score ranges or interpretations. Often, not enough detail was provided to understand what was done exactly, compromising the extent to which findings can be compared across studies. Second, the publishers made small updates to the MBI-22 over time, particularly in relation to the thresholds used to categorize severity of burnout. Many studies unfortunately did not report which version they used, compromising comparability between studies even in otherwise relatively homogeneous settings. For instance, of the eight studies from India having used the MBI-22 in its recommended form and with categorical reporting of results, three used the 2nd edition threshold, four did not report or allow to elsehow infer whether they used the 1st or 2nd edition thresholds, and one appears to have used a mix of both. Finally, the MBI-22 measures three sub-constructs of burnout, one of which is “reduced personal accomplishment” (RPA). The items intended to measure RPA are reversely phrased, however, so that a high raw score indicates low burnout, unlike for the other two subscales. In a large number of studies, it did not become fully clear whether the authors had reversed RPA scores/proportions so that they are interpretable “in the same direction” as the other two dimensions, or whether they reported original scores. Sometimes, inference from the description of results or discussion was possible, whereas in other cases, both numeric estimates and description left doubt as to whether the authors had or had not reversed scores and/or interpreted results correctly.

Results reporting

Depending on the study aim and population, studies reported estimates of levels of mental health either overall for the entire study sample, or broken down by different sample subgroups. For simplicity, we only assessed the extent to which studies broke down results by two key sample characteristics, namely cadre and gender.

Regarding health worker cadre, 64% of studies included respondents from only one staff category and therefore by design reported estimates by cadre (62% of prevalence studies). Of the remaining 55 studies including mixed samples, 44% reported estimates separately by cadre and 56% provided only overall estimates (among prevalence studies: 51% vs 49%, respectively).

Regarding gender, 9% of studies only estimated mental health levels among one gender (usually female nurses) or had a heavily skewed sample in terms of gender, presumably reflecting the reality in the context (usually predominantly male medical doctors with female doctors below 5%) (7% among prevalence studies). Of the remaining 138 studies, 40% reported estimates separately by male and female participants, whereas 60% only provided overall estimates (among prevalence studies: 48% vs 52%, respectively).

Finally, we assessed the extent to which results were displayed adequately, meaning that they could be read without necessitating guesses and that they did not contain obvious errors ( *quality criterion 8*). This was the case for 93% of all studies, and 91% among explicit prevalence studies.

Overall study quality

From the quality judgements in the nine individual categories presented above, we further calculated an overall quality classification for each study as outlined in Table 4. In order to be classified as of high quality, a study had to report results in a readable manner (quality criterion 9), provide sufficient information to allow for the measurements to be interpreted (quality criteria 6 and 8), and reach satisfactory quality scores on all other criteria combined.

As shown at the bottom of Table 5, only 20% of all studies fulfilled our criteria for high quality. We found the majority of studies as of moderate quality (58%), and 22% as of low quality. Among explicit prevalence studies, the proportion of studies in the high and moderate categories was only marginally higher than among all studies.

We did not observe any trend in study quality over time, nor any striking differences by region.

## Discussion

In the context of the SARS-CoV-2 pandemic, health workers’ work realities and their particular risk of facing mental health issues has quickly risen in public attention and generated unprecedented research efforts. Reviews conducted prior to the pandemic ^5– 9^ suggested that mental health research among health workers in LLMIC is limited in quality, albeit without providing clear information on methodological strengths and weaknesses. We therefore used the evidence identified by one of the systematic reviews ^9, 11^ to conduct a detailed quality assessment with the aim of distilling recommendations for future research.

As our review title implies, beyond its limitations in scope – research is concentrated in few countries, on hospital settings in urban centers, and on burnout – our assessment confirmed various quality issues in most of the pre-pandemic body of literature, limiting what can be learned from it.

In particular, we identified major issues with regards to sample representativeness, validity of measurement tools in the respective context, and provision of key information necessary to interpret the numeric figures provided by the authors. In consequence, there are major doubts as to the robustness, interpretability, and external validity of the majority of available studies. Interestingly, studies with an explicit aim to establish prevalences, which we would have expected to receive higher quality scores given that our quality criteria were tailored to this type of study, did not perform better than studies which produced estimates of mental health levels as a by-product.

Some of the identified quality issues can easily be overcome by improved reporting, and we will provide recommendations to this extent below. In terms of reporting, it is interesting to note that a separate analysis of journal quality performed on a subset of the studies revealed that a significant proportion were published in journals likely to be predatory, with questionable peer review processes and editorial quality standards ^24^. Considering that most studies appear to have been led or exclusively conducted by research teams from the respective LLMIC, which is a welcome finding in light of recent increased calls for more equitable global health research structures and partnerships ^25– 27^, this highlights previously expressed needs to financially enable researchers from LLMIC to publish in high-quality journals ^28^, which would arguably increase reporting quality and thereby enhance interpretability and usefulness of research findings.

Other issues are potentially more difficult to address. Issues of representativeness likely necessitate improvements and increased effort in sampling, which however tends to be more resource intensive. Perhaps most importantly, the issue of lack of local validation of measurement tools urgently necessitates substantially more methodological research, as also highlighted by a 2016 systematic review ^29^. Even assuming the cross-cultural validity of “Western” concepts of mental health and illness as such, which is debated ^30^, measurement tools cannot simply be assumed to measure the same constructs across cultural contexts. This issue pertains to both the items and answer options themselves, but even more importantly to the thresholds used to categorize respondents into severity of illness categories. Studies comparing self-reported screening tools to the “gold standard” of clinician-led diagnostic interviews have clearly shown that appropriate thresholds vary substantially between different study populations ^31– 33^. Even in high-income settings where self-reported measures tend to be comparatively well validated and calibrated, lack of accuracy in comparison to clinical interviews has been found a major issue ^34, 35^. Of note, while cultural differences might be most important, there might well be differences in measurement properties between different sub-population within a defined cultural context, for instance between generally well-educated health workers and the likely less educated general or patient population. Unless tools and thresholds are therefore robustly validated in the respective context, interpretation of results remains difficult and speculative, and comparison of study results between contexts close to impossible.

Our study did not investigate whether high-quality evidence regarding the culture-specific validity of the used tools exists, but we focused only information and references provided by the included articles. However, as less than 10% of articles provided information which we judged as convincing, we are confident in concluding that urgent investment into validation studies is necessary. Beyond conducting specific validation studies, authors conducting substantive research can build validation elements into their studies, for instance by advancing the main data collection with a qualitative pre-study or expert assessment of the tool, by collecting additional data for criterion validation, or by performing psychometric analysis of the data. However, our finding that half of the studies made no mention of validity at all, combined with almost none of the studies discussing issues of validity in the study limitations, indicates that capacity building in this area is urgently necessary. 

We did not include in our study the admittedly impressive additional body of literature generated ad hoc in the context of COVID. While it will be interesting to expand the analysis presented in this paper to the COVID-related literature, it appears unlikely that the main messages will change. Specifically, we have no reason to believe that quality of the COVID-related studies is substantially different from what has been done before – likely rather the opposite, given the speed at which pandemic-related research was rolled out.

### Recommendations for future research

Based on the above-presented findings, in order to develop a more comprehensive understanding of the mental health situation among health workers in LLMIC, we urge research funders to make funding available for research with an explicit focus on validating robust methods of estimating mental health prevalence in a variety of socio-cultural contexts, as well as for strengthening comprehensive mental health research capacity, integrating psychiatric, psychological, psychometric, and epidemiological perspectives.

We urge researchers to

- Invest in strong sampling designs likely to lead to representative study samples;
- Invest in culture-specific validation of measurement tools, both as stand-alone projects and within substantive research, by building in psychometric elements into studies (e.g., qualitative pre-studies or expert validation, additional measures for criterion validity) and performing psychometric analysis on data sets (e.g., factor analysis, measurement invariance testing when comparing different sample groups);
- In designing research and writing up study findings, consider the elements summarized in Table 7 to facilitate identification, interpretation, and comparison;
- Aim for publication in reputed journals with high-quality editorial and peer review processes.

**Table 7.** Recommendations for study design and reporting of results.

	Design	Reporting
**Basics**		• Study aim and hypotheses (if applicable) • Year of data collection • Country, geographical region within country • Urban vs rural setting, single- vs multi-site, number of health facilities • Label your study as a mental health study and choose key words that will lead to inclusion in geographic and health workforce MeSH terms etc. in search engines
**Study population**	• Obtain information on total study population size and characteristics	• Level of care, public vs private sector • Health worker cadres • Any other inclusion or exclusion criteria, including rationale • Total study population size (if available)
**Sampling**	• Maximize representativeness • Consider absenteeism due to mental illness • Plan for intended subgroup analysis/ adjustment	• Sampling strategy, intended sample size, rationale for sampling strategy and size • Response rate • Any other information on sample representativeness (e.g. differences in demographic characteristics between study population and sample)
**Reporting of sample ** **characteristics**		• Total sample size • Sample size by key sample characteristics (e.g. cadre, gender, age/seniority group) • Sample characteristics (suggested minimum set: cadre, gender, age, marital status, years of work experience, work hours per week)
**Reporting of ** **outcomes**		• Mental health outcome, including definition where potentially ambiguous
**Outcome measure**	• Consider feasibility of a clinical interview • Consider existing research and validity evidence available in the context when deciding on the outcome measure • Consider measuring additional mental health outcomes for criterion validation	• Outcome measure, including exact name and version (if multiple), reference to tool manual and development/validation paper • Information on language/translation and any modifications • Number of items, item examples, exact response scale including number of response options and numeric codes; if non-standard tool, provide in full • Composite score calculation, including how raw scores were combined, resulting theoretical score ranges and their direction, and thresholds used to classify respondents (if applicable) • Any existing reliability and validity information from the context
**Analysis**	• Consider which reliability and validation analyses are possible with the data (Cronbach’s alpha, factor analysis, measurement invariance testing, criterion validation) • Where robust validity evidence not available: consider performing analyses using multiple thresholds for classification of participants • Consider performing both tests of association and difference, and both bi- and multivariate analysis • Categorize in consideration of the literature which you would like to compare your results to	• Information on reliability and validity analyses • Information on adjustments for sample composition
**Reporting of results**		• Overall and by subgroup, including means/ proportions (ideally both for continuous variables)
**Interpretation**		• Report and discuss implications of methodological study limitations, in particular in regards to sample representativeness and measurement tool validity • Be careful to postulate strong prevalence estimates and with the use of the word “prevalence” in the absence of use of “gold standard” measures and well validated screening tools

### Methodological considerations and recommendations for future updates and reviews

Our review must be read and interpreted in light of certain methodological considerations. First, although we believe to have generated a comprehensive overview of the available literature up to the end of 2020, we cannot exclude the possibility of inadvertently having missed a few relevant studies, for instance by not having included additional databases such as CINAHL potentially listing journals not listed in the chosen databases, and by not adding specific search terms for syndromes/diagnoses beyond the most frequent ones, such as substance use disorder, bipolar disorder, adjustment disorder, physiological disturbances, and (attempted) suicide. We have also only reviewed the academic literature, and thereby omitted any available grey literature. In this context, we would like to briefly comment on why we believe to have found such as substantially higher number of articles than the prior reviews have, but why we still failed to identify all relevant articles through our initial search algorithm. In part, this is of course due to differences in geographic scope and inclusion and exclusion criteria. However, we believe that suboptimal phrasing of titles, abstracts, and key words in the current body of literature also plays a major role. A substantial number of articles, for instance, did not report the country name in the title, but rather the name of a region or city, if anything. Further, rather than using generic terms for the study subjects, such as “health worker” or “healthcare professional”, many studies used the specific terminology in their respective setting, increasing the risk that studies are not picked up even by a very carefully crafted search strategy. Similarly, beyond key standardized syndromes such as burnout and depression, univocal terminology to describe poor psychological wellbeing or pathological forms of stress does not exist, increasing the risk of studies not being found. This issue was likely compounded by the above-discussed journal quality issue. Quite a number of the included studies were published in journals not indexed in the major data bases and therefore not benefitting from MeSH terms and similar concepts. Beyond hoping for improved reporting in the future, we therefore urge researchers planning future reviews to invest time and care into their search strategies in consideration of the above so as to pick up a maximum of relevant research. Finally, we only reviewed the literature published until the end of 2020, thereby omitting the large number of recently published studies having assessed mental health in the context of the pandemic. However, as explained above, we have no reason to believe that this invalidates our main messages.

Second, given the unexpected large amount of studies, we were unable to perform full double screening, data extraction, and quality assessment. Although we took precautions by intensively testing screening procedures, data extraction tools, and quality criteria, we cannot fully exclude certain omissions or errors. Third, we only included articles in English and French language, excluding any relevant literature in other language. In light of the above-discussed high level of “Southern-led” research as well the fact that we did not find any studies from the WHO European Region and Region for the Americas, it may well be that pertinent research published in relevant local languages is not captured by our research. It would therefore be interesting to expand the review to include further languages such as Spanish, Russian, and potentially some South-East Asian languages. 

Finally, we only described study quality, but did not extract data to allow understanding the drivers of heterogeneity in study quality. Such an understanding of predictors of poor study quality will be of key importance in tailoring systematic quality improvement efforts, beyond our generic recommendations. Future updates might for instance want to look at type/ level of study funding and at the research team’s level of experience as likely important predictors of study quality.

## Conclusion

Our study shows that the pre-pandemic body of literature on mental health of health workers in LLMIC, while rather impressive in size, is limited in what we can learn from it by shortcomings in methodological quality. In particular, we identified major issues with regards to sample representativeness, validity of measurement tools in the respective context, and provision of key information necessary to interpret the numeric figures provided by the authors. We urge funders to invest in validation research as well as in mental health research capacity building. We encourage researchers to do the same, and to further improve on methodological quality of research and on reporting of methods and findings.

## Data Availability

No data are associated with this article. LSHTM Research Online: Additional Files: "Much research, but little learned to date: A scoping review of the methodological quality of research on mental health of healthcare professionals in low- and lower-middle income countries." https://doi.org/10.17037/PUBS.04665736 ^16^. This project contains the following extended data: Additional Files 1: Completed PRISMA-ScR checklist (Scop-rev_MHHW_220523_AddF_1.docx) Additional Files 2: List of low- and lower-middle income countries (LLMIC) as per the World Bank’s 2019 classification (Scop-rev_MHHW_220523_AddF_2.docx) Additional Files 3: Overview and references of included studies (Scop-rev_MHHW_220523_AddF_3.docx) Data are available under the terms of the Creative Commons Attribution 4.0 International license (CC-BY 4.0).
