# Supplementary material for: A scoping review of the methodological quality of research on mental health of healthcare professionals in low- and lower-middle income countries
Source: Wellcome Open Res. 2023 Jul 14;7:169. Originally published 2022 Jun 13. [Version 2] doi: 10.12688/wellcomeopenres.17916.2 (PMC10442593; doi:10.12688/wellcomeopenres.17916.2)
Supplement: Peer review history

# Peer review: Reviewer response for version 2

**DOI:** 10.21956/wellcomeopenres.21802.r62944

**Published:** 2023-08-21

**Authors:** Shuyan Liu

**Affiliations:** 1 Department of Psychiatry and Psychotherapy, Charité – Universitätsmedizin Berlin, Berlin, Germany

I would like to approve the current revised article, but there may be a need to update the search. The search is more than one year old (by the end of 2020). Ideally it should be updated.

Are the rationale for, and objectives of, the Systematic Review clearly stated?

Partly

Is the statistical analysis and its interpretation appropriate?

Not applicable

Are sufficient details of the methods and analysis provided to allow replication by others?

Partly

Are the conclusions drawn adequately supported by the results presented in the review?

Yes

Reviewer Expertise:

NA

I confirm that I have read this submission and believe that I have an appropriate level of expertise to confirm that it is of an acceptable scientific standard.

# Peer review: Reviewer response for version 2

**DOI:** 10.21956/wellcomeopenres.21802.r62943

**Published:** 2023-08-21

**Authors:** Marcia Scazufca

**Affiliations:** 1 Departamento de Psiquiátria e LIM-23, Hospital da Clínicas, Faculdade de Medicina, Universidade de Sao Paulo, São Paulo, State of São Paulo, Brazil

The authors answered my questions adequately and made the necessary changes to the manuscript, including the title. I have no further comments about the manuscript.

Are the rationale for, and objectives of, the Systematic Review clearly stated?

Partly

Is the statistical analysis and its interpretation appropriate?

Not applicable

Are sufficient details of the methods and analysis provided to allow replication by others?

Partly

Are the conclusions drawn adequately supported by the results presented in the review?

Partly

Reviewer Expertise:

Mental health, primary care, older adults

I confirm that I have read this submission and believe that I have an appropriate level of expertise to confirm that it is of an acceptable scientific standard.

# Peer review: Reviewer response for version 1

**DOI:** 10.21956/wellcomeopenres.19854.r56115

**Published:** 2023-05-03

**Authors:** Marcia Scazufca

**Affiliations:** 1 Departamento de Psiquiátria e LIM-23, Hospital da Clínicas, Faculdade de Medicina, Universidade de Sao Paulo, São Paulo, State of São Paulo, Brazil

The article assesses the quality of research on the mental health of health professionals in the period leading up to the onset of the pandemic of COVID-19 in LLMIC. Nine criteria were used to assess the articles' quality, including eight items on aspects of the studies' methodology and one item about reporting (Table 3). Thus, the evaluation of the quality of the studies did not consider factors that certainly impact the methods of the studies, which are whether the study received funding/type of funding and the research team's experience. This information may provide subsidies for the discussion of the results already presented.

 I agree with the comments made by Shuyan Liu (reviewer). Below are my comments on the paper:

**Title. **"Much research, but little learned to date".

 The authors considered that there is much research on this topic in LLMIC and that little is learned from these studies. The conclusion about this "quantity" should be based on the need for studies in this area in LLMIC and on comparisons with other countries not included in this review. My impression (not based on data) is that there is little research, that studies are restricted to a few settings ("Close to two-thirds of studies were conducted in four countries"), and that efforts are enormous to produce analyses of some quality given the difficulty of funding and having experienced research teams in LLMIC. I suggest revising the title.

**Method:**

1. Concept: Regarding the inclusion of other psychiatric diagnoses, in addition to the diagnoses suggested by Shuyan Liu, the review could have included studies on suicide/suicide attempts in this population, as these events are often associated with mental disorders. 
2. Context. "..... clinically qualified health care staff working in all LLMIC care settings worldwide by the end of 2020." What is the rationale for not including healthcare staff from middle-income countries?
3. Extracted data: studies were assessed using nine quality criteria. Why did the authors not include quality criteria not intrinsically associated with the research methodology? For example, authorship (experience, seniority of the authors, if they are authors of other poor/good quality studies included in the review), and if the study received funding (type of funding: scholarship or grant for the study, etc.).

The pandemic highlighted the importance of professionals' mental health to the healthcare system's functioning and the quality of care. Knowing which elements outside the control of researchers influence the quality of studies is helpful information for research agencies, health systems and researchers, among others, and for developing future interventions to reduce mental distress in health workers.

**Discussion:**

1. Publishing in journals that may be predatory: The authors suggested: "financially enabling LLMIC researchers to publish in more reputable journals". This is a somewhat simplistic way of solving the problem. The publication of a paper is the result of a process that begins with developing a project. The difficulties that arose during this process will hardly be solved by sending these articles to better-quality scientific journals.
2. "As noted, all but two studies used self-report to measure the mental health outcome, rather than the gold standard of a clinician-led diagnostic interview". I doubt that this reason (use of self-report assessments) is among the most relevant for these studies’ poor or lack of quality. Self-report assessments (Beck depression, PHQ-9, DASS-21, etc.) have been used worldwide in prevalence studies and clinical trials. Suppose authors want to use this argument to justify the poor or lack of quality; it is essential to present references showing that these scales are unsuitable for prevalence studies.
3. The suggestion “we urge funders to invest in validation research as well as in mental health research capacity building” is appropriate but could be based on data from the studies included in the review (whether or not they received funding and the relationship with the quality of the studies).

Are the rationale for, and objectives of, the Systematic Review clearly stated?

Partly

Is the statistical analysis and its interpretation appropriate?

Not applicable

Are sufficient details of the methods and analysis provided to allow replication by others?

Partly

Are the conclusions drawn adequately supported by the results presented in the review?

Partly

Reviewer Expertise:

Mental health

I confirm that I have read this submission and believe that I have an appropriate level of expertise to confirm that it is of an acceptable scientific standard, however I have significant reservations, as outlined above.

# Response

**Published:** 2023-06-28

**Authors:** Julia Lohmann

**Affiliations:** London School of Hygiene & Tropical Medicine, UK

Many thanks for your insightful comments. In response, we have made the following changes:

- Comment #1 regarding explanatory factors of quality: We did indeed not attempt to extract information potentially explaining variation in study quality, but only aimed to describe. Following your comment, we have considered including some factors potentially explaining heterogeneity in study quality. However, very few of the included articles include relevant information about authors and funding. Finding respective information online or contacting authors appears difficult within reasonable effort. We therefore abandoned the attempt, but have added a paragraph to the end of the methodological considerations as a note for future research. 
- Removed the first part of the title sentence
- Included (attempted) suicide in the methodological considerations alongside the other diagnoses/syndromes suggested by the other reviewer. If framed as a mental health issues, however, our search should have picked up on them. 
- Slightly revised the discussion section on three counts:
  - Made clear that the paragraph pertaining to our hope of higher-quality journals solving some quality problems only relates to the dimension of reporting, not to other quality issues as you rightfully highlight. 
  - Rearranged and toned down the point on clinician-led interviews instead of self-reported screening tools. Indeed, the paragraph had been added as the result of a prior round of reviews in a different journal. Two (cited) studies (Levis et al, 2020; Thombs et al, 2018) do show rather dramatic differences in prevalence estimates between diagnostic interviews and self-reported tools, calling them into question fundamentally, despite wide use. However, we tend to agree with you that this is not the most pressing measurement issue and we would not want to imply this as a killer argument to justify poor quality stemming from other issues.

Finally, on your question regarding middle-income countries: For the past ~5 years, it has become common practice to differentiate the middle income country classification into upper- and lower-middle income countries, to account for the dramatic variation of health systems in the middle income category. We have followed this emerging standard and focused only on low-income and lower-middle income countries.

# Peer review: Reviewer response for version 1

**DOI:** 10.21956/wellcomeopenres.19854.r54844

**Published:** 2023-03-17

**Authors:** Shuyan Liu

**Affiliations:** 1 Department of Psychiatry and Psychotherapy, Charité – Universitätsmedizin Berlin, Berlin, Germany

This paper examines research quality in healthcare professional’s mental health in low- and middle-income countries. While there is a surfeit of studies assessing the mental health of healthcare professionals, the current study takes a systematic approach by evaluating their qualities. Such findings are a valuable complement to the existing mental health literature in low- and middle- income countries.

 The following are aspects of the paper which could benefit from minor corrections or clarifications:

1. When talking about “much research, but little learned to date” in the title, I would recommend a written clarity.
2. In the abstract, the rationale for the research before pandemic should be further justified. The literatures were identified from when to the end of 2020. Why did the authors include the literatures only in English or French language? What were the keywords that used for searching literature? What are the key study characteristics? How did the authors evaluate the study quality? Why was burnout chosen as the main mental health outcome? It was unclear if each quality criteria had certain weights. What is added value of those 20% of “high quality” studies? Any reports on power, effect sizes and sample size estimation? What does “its impressive size” mean?
3. In the introduction, I would recommend including the findings of previous studies on mental health of health worker, quality assessments and available evidence in LLMIC.
4. In the concept, did the authors include substance use disorder, bipolar disorder, adjustment disorder, and physiological disturbances (e.g., insomnia)?
5. Regarding study population, why were psychiatrists, psychotherapists, psychological consultants, and clinical social workers not being considered?
6. As the representativeness of samples and validity of measurement tools are the key results, these could be incorporated into a table or visualise it.

Are the rationale for, and objectives of, the Systematic Review clearly stated?

Partly

Is the statistical analysis and its interpretation appropriate?

Not applicable

Are sufficient details of the methods and analysis provided to allow replication by others?

Partly

Are the conclusions drawn adequately supported by the results presented in the review?

Yes

Reviewer Expertise:

NA

I confirm that I have read this submission and believe that I have an appropriate level of expertise to confirm that it is of an acceptable scientific standard, however I have significant reservations, as outlined above.

# Response

**Published:** 2023-06-28

**Authors:** Julia Lohmann

**Affiliations:** London School of Hygiene & Tropical Medicine, UK

Many thanks for the helpful comments. In response, we have made the following revisions in the updated version of the manuscript:

- Removed the first part of the title to retain only "A scoping review ... "
- Slightly amended the abstract, but were unable to include detail for most of your questions due to the 300 word limit; all information is included in the main text, however. The rationale for including research prior to the pandemic is that the review was conducted prior to the pandemic; this article makes secondary use of the articles identified by a systematic review.
- Added detail on the conclusions of prior reviews on study quality; we have not added detail on substantive findings of prior reviews as this is beyond the focus of this manuscript, and as we do not fully concur that substantive findings are always valid due to the highlighted quality concerns.
- Added to the limitations that search terms for certain syndromes/diagnoses were not included. Specifically, we did not  include bipolar disorder and adjustment disorder which, while certainly relevant to psychiatric/general population-focused research, do not appear relevant to the specific population of health workers, for which studies tend to take a work psychology / positive psychology lense on mental health. We did consider including substance use disorder and physiological disturbances. Similar to stress, however, we struggled with drawing the line – for instance, a number of studies reports on drinking or sleeplessness among health workers, but doesn’t necessarily frame the issues as mental health issues, with clear and standardized definition of when respective behavior is indicative of a disorder in a clinical sense, see respective sentence in the “Study selection” section of the Methods. We therefore did not include specific search terms and did not consider respective studies, unless they clearly framed and defined the listed disorders as a mental health issue.

We did not make any changes in relation ti your last two comments, but would like to give a brief explanation:

- *Why were psychiatrists, psychotherapists, psychological consultants, and clinical social workers not being considered? *They were considered and included. However, given the generally very low availability of these cadres in LLMIC settings, we did not include specific search terms for the professions as tests during the search strategy definition showed that this would have further inflated the number of irrelevant search results. This is due to the fact that any (irrelevant) paper on (general or specific) population mental health mentioning the need for treatment by a mental health specialist in the discussion section of the abstract would have been picked up, which is already an issue with the generic health worker terms. However, any studies specifically on these cadres would have been picked up by the generic and MeSH terms for health workers/healthcare personnel.
- *As the representativeness of samples and validity of measurement tools are the key results, these could be incorporated into a table or visualise it.* These are included in Tables 5 and 6. However, we would like to point out that we did not assess in detail the representativeness of the samples – but rather whether the information provided allowed for a reasonable judgement. Most articles did not provide tangible information, but rather barely enough information for an informed guess on whether the sample might be representative for the respective setting. Similarly, we did not assess whether the tools used were indeed validated in the specific setting, but rather whether the study reported on it. 
